# Type I Interferon Induction and Exhaustion during Viral Infection: Plasmacytoid Dendritic Cells and Emerging COVID-19 Findings

**DOI:** 10.3390/v13091839

**Published:** 2021-09-15

**Authors:** Trever T. Greene, Elina I. Zuniga

**Affiliations:** Division of Biological Sciences, University of California, San Diego, CA 92093, USA; ttgreene@ucsd.edu

**Keywords:** IFN-I, plasmacytoid dendritic cells, viral infection, LCMV, influenza, HIV-1, HCV, HBV, COVID-19, SARS-CoV-2

## Abstract

Type I Interferons (IFN-I) are a family of potent antiviral cytokines that act through the direct restriction of viral replication and by enhancing antiviral immunity. However, these powerful cytokines are a caged lion, as excessive and sustained IFN-I production can drive immunopathology during infection, and aberrant IFN-I production is a feature of several types of autoimmunity. As specialized producers of IFN-I plasmacytoid (p), dendritic cells (DCs) can secrete superb quantities and a wide breadth of IFN-I isoforms immediately after infection or stimulation, and are the focus of this review. Notably, a few days after viral infection pDCs tune down their capacity for IFN-I production, producing less cytokines in response to both the ongoing infection and unrelated secondary stimulations. This process, hereby referred to as “pDC exhaustion”, favors viral persistence and associates with reduced innate responses and increased susceptibility to secondary opportunistic infections. On the other hand, pDC exhaustion may be a compromise to avoid IFN-I driven immunopathology. In this review we reflect on the mechanisms that initially induce IFN-I and subsequently silence their production by pDCs during a viral infection. While these processes have been long studied across numerous viral infection models, the 2019 coronavirus disease (COVID-19) pandemic has brought their discussion back to the fore, and so we also discuss emerging results related to pDC-IFN-I production in the context of COVID-19.

## 1. Introduction

The Type I interferon (IFN-I) family are antiviral and antineoplastic cytokines critical for the control of most types of viral infection [1,2]. This family includes 13 subtypes of IFN-α in humans (14 in mice) one IFN-β as well as a handful of other gene products (reviewed in [2]). While all cell types can produce IFN-I, plasmacytoid (p) dendritic cells (DCs) produce IFN-I and other interferons at exceptional levels, including all 13 subtypes of IFN-α, IFN-β, and 3 subtypes of IFN-λ, and IFN-τ (reviewed in [3,4,5] and described in [6]). As such, pDCs promote control of multiple types of viral infection (reviewed in [4]). However, despite the critical importance of IFN-I to control viral infections the consequences of IFN-I are not exclusively beneficial. Excessive and/or late IFN-I is associated with increased mortality in several animal models of viral infection (e.g., Influenza [7], arenavirus mediated hemorrhagic fever [8], MERS and SARS-CoV-1 infections in mice [9,10]). Furthermore, persistent IFN-I signaling in a mouse model of chronic viral infection promotes T cell exhaustion and compromises viral control [11,12]. Although it is notable that the addition of recombinant IFN-I early in the same infection promotes viral clearance [13]. Finally, aberrant IFN-I signaling is a signature of many autoimmune disorders several of which have been associated with activation of pDCs (reviewed in [14]).

Despite the importance of pDC derived IFN-I for the control of many viruses, after their initial activation pDCs rapidly lose their capacity to produce these antiviral mediators, a state that we refer to as “pDC exhaustion” (Reviewed in [15]). While this pDC exhaustion can favor prolonged viral replication and opportunistic secondary infections [16,17,18], it may have evolved as a “default” behavior to avoid the potentially harmful effects of sustained IFN-I production. Additionally, this adaptation may also help protect against interferonopathies in the absence of infection.

Here we provide a review of the available literature on pDC IFN-I induction and the mechanisms that support and oppose this important function of pDCs. First, we describe the pathways that promote or suppress pDC IFN-I production on a per-cell basis including modulators of signaling, inhibitory receptors, cytokines, and metabolism. Afterwards, we describe mechanisms that reduce or enhance pDC numbers. Together both types of regulation help to determine the availability of pDC derived IFN-I upon a viral infection.

The COVID-19 pandemic caused by SARS Coronavirus-2 (SARS-CoV-2) has led to rapidly evolving investigation of this disease. These studies have revealed that many of the features of other acute and potentially deadly infections are conserved in COVID-19, including critical roles for IFN-I in both protection and disease. Furthermore, COVID-19 has been associated directly with suppression of pDC IFN-I on a per cell basis [19], as well as with reduced pDC numbers [19,20,21,22]. At the end of this review, we describe emerging literature on pDCs, IFN-I, and COVID-19 and discuss how this relates to the established framework in the field. We will avoid a full review of pDC development, the roles of pDCs in tolerogenic responses, and the role of pDC IFN-I production in cancer. We suggest the reader consults these recent reviews as resources for those topics [5,15,23,24].

## 2. pDC IFN-I Production after Viral Infections

### 2.1. Uptake of Nucleic Acids

The first step in recognition of a viral infection by a pDC is the uptake of nucleic acids, and their delivery to toll-like receptor (TLR) containing endosomes. While the uptake of stimulatory oligonucleotides used to study pDC function in vitro primarily occurs through clathrin mediated endocytosis [25], viral material uptake can reach the TLR-containing endosomes through endocytosis or autophagy [26,27]. Furthermore, the viral material sensed can be functional or defective viral particles [28,29], exosomes [30], and even productive infection and replication within the pDC themselves [26]. Additionally, Fc receptors can facilitate uptake of immunocomplexed nucleic acids into pDCs [31] and this can trigger their IFN-I production [32]. Indeed, this process has been implicated in abnormal IFN-I elevation in systemic lupus erythematosus (SLE) [32,33]. In contrast, FcγIIB-based internalization of immune complexes has been shown to oppose IFN-I production in pDCs, and this is associated with reduced IFN-I production in response to Sendai virus (SV) infection when virus-specific-antibodies are present, suggesting this route could exist as a way to quell IFN-I responses [31]. Recently, it has also become well-established that, in some cases, pDCs require cell-cell contact with infected cells to robustly produce IFN-I [34,35,36,37,38,39,40,41], and recent observations in vitro support the idea that an “interferogenic synapse” can develop between pDCs and infected cells [42]. How stimulatory material is exchanged in this synapse, and its potential function in vivo still needs further study. Ultimately, the precise route for nucleic acid uptake depends on the type of viral infection and context, and this route can influence the magnitude of pDC IFN-I responses [31,34].

### 2.2. Signaling by TLR7/TLR9

The primary mechanism by which pDCs are known to recognize exogenous and endogenous RNA and DNA is through TLRs 7 and 9, respectively. These TLRs are highly expressed in pDCs and stimulation of either can lead to high levels of IFN-I production (Reviewed in ref [43]). TLR7 and TLR9 signal through a combination of MyD88-NF-kB and MyD88-IRF7 pathways (Reviewed in refs [44,45]). The relative use of these signaling pathways depends on the subcellular compartments in which the specific TLR is located and varies with the stimulus [46,47,48]. For example, the multimeric oligonucleotide family of CpG-A localizes to early endosomes where they preferentially activate the MYD88-IRF7 pathway inducing high levels of IFN-I [46,47]. In contrast the monomeric oligonucleotide family of CpG-B localizes to an endolysosomal compartment [46,47]. In the last-mentioned case the MYD88-NF-kB pathway is preferentially activated and leads to production of pro-inflammatory cytokines such as tumor necrosis factor alpha (TNFα) [46]. In pDCs, but not in conventional (c) DCs, the trafficking of CpG-A to early endosomes and their cytokine production relies on signaling through Src kinases Lyn and Fyn [49]. This connection between differential trafficking and signaling has been used to explain the long-standing observation that CpG-A induces higher levels of IFN-I, while CpG-B induces more pro-inflammatory cytokines [50,51]. It is important to note that neither of these cases is absolute as CpG-B induces some production of IFN-I while CpG-A also induces pro-inflammatory cytokines.

### 2.3. TLR2/12

While most of the work in pDCs focuses on TLR7 and TLR9, pDCs can also express TLR2 and TLR12 [52,53]. As with TLR7 and TLR9, recognition of *Toxoplasma gondii* profilin by TLR12 is sufficient to stimulate pDCs to produce IL-12. These observations, along with the observation that TLR12 deficient mice are more susceptible to *T. gondii* infection than wild type animals, suggest a potential role for pDCs in host resistance to this pathogen [52]. However, not all TLRs expressed in pDC stimulate the production of IFN-I or pro-inflammatory cytokines. Indeed, the commensal associated molecule polysaccharide A (PSA), which is sensed via TLR2 [54], has been shown to drive an immunoregulatory phenotype in pDCs that protects mice against 2,4,6-trinitrobenzenesulfonic acid (TNBS) induced colitis [53]. Further research into the roles for these and other TLRs expressed by pDCs are an exciting avenue of future work and will provide deeper insight into how pDC responses are regulated in a variety of contexts.

### 2.4. Cytosolic Nucleic Acid Sensors

In addition to TLRs pDCs express several cytosolic sensors of nucleic acids, some of which have been established to induce pDC activation and IFN-I production. The cytosolic nucleic acid receptors DHX36 and DHX9 have been shown to bind CpG-A and CpG-B and induce IFN-I and pro-inflammatory cytokine production, respectively [55]. On the other hand, it has been proposed that the cytosolic sensor retinoic acid-inducible gene I (RIG-I) does not induce IFN-I production in pDCs. This assertion is based on IFN-I levels in response to Newcastle disease virus (NDV), which did not change in RIG-I deficient pDCs [56]. However, it has since been shown that in some cases RIG-I can contribute to IFN-I production in pDCs. Specifically, adaptors of RIG-I help drive IFN-I production in pDCs in response to NDV in the absence of IFN-I restriction of viral growth [57]. Additionally, RIG-I may drive pDC responses to the cell-free yellow fever live vaccine (YF-17D) [34]. Though notably, the amount of IFN-I produced as the result of RIG-I signaling is less than that produced in response to TLR7 signaling [34].

Recently the cyclic GMP-AMP synthase (cGAS)-stimulator of IFN genes (STING) pathway has also emerged as a method by which pDCs can recognize and respond to insult. In this pathway the enzyme cGAS recognizes cytosolic dsDNA and makes cyclic GMP-AMP (cGAMP). Cyclic dinucleotides can then be recognized by STING which induces IFN-I expression (Reviewed in ref [58]). Human pDCs express both cGAS and STING and can produce IFN-I in response to both cytosolic dsDNA as well as the STING ligands cGAMP, dAMP, and diGMP [59]. STING stimulation of IFN-I in human pDCs associates primarily with nuclear translocation of IRF3 (in contrast to IRF7 for TLR7 and TLR9) [59,60]. Importantly, potentially as a result of these differences, stimulation with cGAMP induces significantly lower levels of IFN-α, IFN-β, and IFN-λ, that is also produced with slower kinetics, as compared to CpG-A stimulation of TLR9 [60]. Furthermore, STING pre-activation reduces pDC IFN-I production in response to TLR9 ligation [60].

## 3. Suppression of pDC IFN-I Production after Viral Infections

### 3.1. pDC Functional Exhaustion

Within a few days after an in vivo viral infection the capacity of pDCs to produce IFN-I is severely reduced [16,61], a phenotype that we now refer to as pDC exhaustion. This was first described after acute and chronic LCMV infection in mice, and coincided with a dramatic attenuation of systemic IFN-I early after its peak at day one post infection [16]. pDC exhaustion was also shown to associate with compromised Natural Killer cell activation, Interferon-γ production and cytotoxicity in response to an unrelated secondary virus [16]. Lee et al. confirmed these findings, and additionally showed pDC exhaustion associated with deficient cross-priming of T cells to an unrelated antigen [61]. Importantly, the pDC exhaustion phenotype described in vivo is consistent with other studies showing that pDCs from patients infected with HIV [62,63,64,65], HCV [66,67,68,69,70] and HBV [71,72,73] as well as macaques infected with SIV [74,75] exhibited reduced per-cell production of IFN-I when re-stimulated ex vivo. Additionally, in HIV-infected humans systemic IFN-I is attenuated early after infection [76], and reduced pDC numbers and/or loss of their ex vivo IFN-I production are correlated with opportunistic infections [18,77,78,79]. Remarkably, this is true even when considered independently of CD4 T cell numbers [77]. It is additionally important to note that IFN-I exhaustion in pDCs is sometimes associated with reduced capacity to produce other cytokines such as TNFα [17]. pDC exhaustion, however, is primarily studied in the context of IFN-I and it is unclear whether other interferons that can also be produced by pDCs (i.e., IFN-λ, and IFN-τ) are likewise suppressed in this condition.

More recently, it has been established that, analogous to T cell receptor (TCR) induction of CD8 T cell exhaustion, in vivo pDC exhaustion in LCMV-infected mice is dependent on TLR7, which reduces the expression of E2-2, a transcription factor essential for pDC development and function [80], via cell-intrinsic signaling in pDCs [17]. Evidence from HIV infection also suggests that persistent stimulation may promote pDC exhaustion as ex vivo IFN-I production by pDCs is reduced in patients with high viral load [62,63], recovers during the administration of successful antiretroviral therapy [65], but becomes suppressed again upon interruption of antiretroviral treatment [63]. Additionally, E2-2 expression is reduced in pDCs from HIV viremic patients [17], and in a human pDC cell line when stimulated with TLR ligands for 2 days [81]. Taken together, these data suggest that persistent TLR engagement may promote pDC IFN-I exhaustion in mice and human pDCs, analogous to what has been described for CD8 T cell exhaustion after chronic stimulation through the TCR [82].

### 3.2. Inhibitory Receptors

IFN-I production in pDCs can be modified by receptors expressed on their surface. Diverse families of receptors such as BDCA2 [83], Siglec-H [84], ILT7 [85], FcεRI [86], DCIR [87], Tim-3 [64], CD28 [88], E-cadherin [89], and Nkp44 [90] all have established rolls in modulating pDC cytokine production downstream of ligation. Much of this evidence exists ex vivo and the role of most of these receptors in pDC function during in vivo viral infection is not yet established. Even for the few that have been investigated in vivo little is known. Mice deficient in CD28 have enhanced serum IFN-I and increased IFN-I transcript levels in pDCs after either LCMV or MCMV infection, and this associates with improved control of MCMV [88]. Similarly, Siglec-H deficient mice also have increased systemic IFN-I after MCMV infection [91]. In addition, in HIV patients a functionally distinct subset of pDC expressing Tim-3 has been identified, correlating expression of this marker to distinctly reduced IFN-I production capacity in human infection [64].

As blockade of inhibitory receptors has been an effective treatment strategy for the modulation of immune cell functions, it is tempting to speculate that surface inhibitory receptors could be promising targets for enhancing pDC function. For example, the asthma drug Omiluzimab, which acts by sequestering free IgE, drives downregulation of FcεRI (a receptor for IgE) on mast cells and basophils [92]. However, as a side-effect Omiluzimab treatment also enhances IFN-α production capacity in pDCs [93]. This enhancement associated with reduced frequency of rhinovirus detection in a cohort of asthma patients treated with Omiluzimab [94], suggesting that targeting pDC inhibitory receptors has the potential to improve human anti-viral responses in vivo. However, pDC inhibitory receptors may also provide pathogens routes to modulate pDC function. For example, HIV protein Vpu has been shown to subvert anti-viral pDC functions by redistributing BST2 on infected cells. This permits viral exit while still engaging the inhibitory receptor ILT7 on pDCs to suppress IFN-I production [95]. Thus, the multiple inhibitory receptors expressed by pDCs may offer mechanisms by which both hosts, and pathogens can modulate pDC-derived IFN-I in the context of infections.

### 3.3. Cytokines and Hormones

Many soluble factors can modulate pDC function. Negative regulators of pDC IFN-I production include Transforming Growth Factor Beta (TGFβ), Interleukin (IL)-10, prostaglandin E2 (PGE2), and TNFα [96,97,98,99,100]. While positive regulators include IL-4 [97], IL-7 [97], IL-15 [97], Estrogen [101], and IFN-I itself [97,102,103,104]. As with inhibitory receptors the functions of most of these soluble molecules are primarily established in vitro or ex vivo and the roles these factors play and the mechanisms by which they modulate pDC function during viral infection in vivo are not fully elucidated.

The fact that IFN-I itself enhances pDC IFN-I production is worth noting as availability of autocrine or paracrine IFN-I signaling can enhance the percentage of IFN-I producing pDCs [97,102,103,104], although IFN-I also induces pDC death in HSV-1 infected mice [105]. The role of TNFα is also notable as pDCs also produce TNFα upon TLR [106] or FCεR1 engagement [86,107] and thus this may represent another autocrine and/or paracrine negative feedback loop. Integrating the potential autocrine and/or paracrine effects of IFN-I and TNFα may offer a partial explanation of differences between CpG-B and CpG-A stimulation. For example, it would be predicted that a pDC which starts producing IFN-I would receive autocrine signals that drive it toward further production of IFN-I. In contrast, a pDC producing primarily TNFα would receive feedback that opposes IFN-I production. This has been partially proposed before although these studies considered only the impacts of IFN-I positive feedback [104] and TNFα negative feedback [108,109] in isolation. Further study, and perhaps mathematical modeling may shed light on how to disentangle these feedback loops, as well as the established intrinsic differences in cell signaling, which support this phenomenon.

It is also worth noting the positive impact of Estrogen on pDC function [101]. This is one part of a larger convergence of factors that drive increased pDC responses in people with more than one X chromosome [101,110,111,112,113]. A phenomenon also partially driven by TLR7, which is expressed on the X chromosome but resistant to X chromosome inactivation [114]. Indeed, both X chromosome content and sex hormone levels contribute to increased IFN-I production in mice [111]. Furthermore, a study inclusive of both cis and transgender male and female humans recently demonstrated that both chromosome number and sex hormone levels associate independently with pDC functional capacity in humans as well [115].

### 3.4. Metabolic Requirements in pDCs

IFN-I responses in pDC require large-scale and rapid synthesis of IFN-I mRNA/protein. The metabolic requirements for this process are just beginning to be uncovered. It has been established that pDCs require both Oxidative metabolism (OxPhos) as well as glycolysis to maintain their function [116,117,118]. Furthermore, mammalian target of rapamycin (mTOR), a central regulator of metabolism, is essential for pDC IFN-I production [119]. While multiple studies agree that pDCs adjust their metabolism after stimulation the details of these changes are still being established, Bajwa et al. show that at 24 h post stimulation with influenza, lactate terminal glycolysis is enhanced in pDCs while OxPhos is slightly suppressed [116]. In contrast Hurley et al. show that 6 hr of influenza or HSV exposure drives a slight increase in oxygen consumption rate (OCR) indicating increased OxPhos [118]. It is likely that the choice of distinct time-points in these studies is responsible for the observed differences. Metabolic changes after stimulation may also provide feedback loops that help control and tune down IFN-I production. Indeed, as high levels of lactate can inhibit pDC IFN-I production [120], then it might be expected that over time enhanced levels of lactate terminal glycolysis after stimulation as described in Bajwa et al. [116] could inhibit pDC IFN-I production. Furthermore, Wu et al. show that IFN-I signaling can enhance basal OCR in both pDCs and epithelial cells, and show oxidative metabolism is essential to pDC function [117]. Thus, this could be part of the mechanism by which IFN-I enhances its own production. Further studies will be necessary to work out the precise details explaining how changes in pDC metabolism in response to environmental cues may tune their function. Furthermore, it will be especially important to assess how pDCs adapt their metabolism after in vivo infection.

## 4. Reduction of pDC Numbers after Viral Infections

In addition to the direct suppression of pDC IFN-I production by the above mechanisms pDC-derived-IFN-I is also compromised in many infections by a reduction in pDC numbers. This has been well established for HIV-1 [18,62,63,64,65,121,122], HCV [66,67,68,69,70,123,124,125] and HBV [71,72,73] infections, in humans. Reduced numbers of pDCs have also been reported during SIV infection in macaques [74,75,126,127], and LCMV, CMV, HSV, and VSV in mice [13,16,17,61]. The mechanisms by which pDC numbers may be altered include reductions in development from bone marrow (BM) progenitors, increased apoptosis, changes in pDC proliferative capacity, and conversion of pDCs into conventional (c)DC-like cells, as discussed below.

### 4.1. Compromised pDC Development from Bone Marrow Progenitors

As pDCs are typically short-lived (persisting for only ~3 days in the periphery of C57Bl/6 mice) [128,129] it is expected the entirety of the pDC population will turn over in the course of any infection lasting longer than a few days. However, in non-resolving infections pDC exhaustion and reduced numbers of pDCs are maintained long-term [16]. One possibility is that pDC progenitors are modulated by the infection in a way that they generate fewer and functionally deficient pDCs. Indeed, pDC generation from BM is reduced for long term in mice infected with a persistent LCMV variant, and transiently decreased after infection with an acute LCMV strain [17]. This associates with a numerical reduction in pDC progenitors in the BM, but also extends to a reduced capacity of these progenitors to produce pDCs and reduced expression of E2-2 [17]. Furthermore, the few pDCs generated from BM progenitors from infected mice develop to produce less IFN-I when stimulated ex vivo [17]. Reduced E2-2 in both the progenitors and matured DCs from these cultures raises the possibility that a transcriptional state inherited from progenitors may contribute to the exhaustion phenotype in their pDC progeny. It should, however, be considered as a caveat that virus does persist in these BM cultures and thus may provide persistent TLR7 stimulation to newly generated pDCs driving the observed exhaustion phenotype. Additionally, as advances are made in understanding the development of pDCs, it has become apparent that the BM pDC progenitors analyzed in this study were heterogeneous [130]. Thus, it will now be necessary to distinguish whether a specific depletion of E2-2 high pDC committed progenitors or a general suppression of E2-2 expression across multiple progenitor populations is responsible for these observations.

### 4.2. Apoptosis

Increased levels of apoptosis in pDCs have been observed in HIV and SIV infections in humans and macaques respectively and HSV infection in mice [75,105,126,131]. For HSV-1 infection in mice IFN-I signaling is at least partially responsible for this effect [105], suggesting that IFN-I driven apoptosis is part of a negative feedback loop limiting pDC IFN-I production after infection. It is also worth considering a role for glucocorticoids (GCs), which can be enhanced after viral infection [132,133] and can also promote pDC apoptosis [134,135,136]. However, as TLR stimulation opposes GC induced apoptosis, it is unclear whether increased GC levels during infection would ultimately result in increased pDC apoptosis in vivo and so further study will be needed to evaluate this.

### 4.3. Proliferation

Although during persistent LCMV infection pDC development is compromised, and apoptosis is increased, pDC numbers in peripheral lymphoid tissues are not universally decreased after infection. Indeed, at day 10 after LCMV infection there is an increase in pDC numbers in the spleens of infected mice [17]. This corresponded to an expansion of CD4^−^CCR9^−^ and CD4^−^CCR9^+^ pDC subsets with increased proliferative capacity [17]. While CD4^−^CCR9^−^ and CD4^−^CCR9^+^ pDCs have been described in the BM [128,137,138], where they do exhibit proliferative potential as precursors of CD4^+^CCR9^+^ pDCs, splenic pDCs are predominantly CCR9^+^ at steady state and do not proliferate [128,137,138]. In LCMV infection this gain in proliferative subsets is dependent on both IFN-I and TLR7 signaling, in contrast to functional exhaustion which requires only the latter [17]. Notably, the cell-cycle marker Ki67 has also been reported to be enhanced in pDCs from SIV infected macaques [75,126,127]. As development from the BM is compromised at this time, this pool of proliferating pDC in the spleen (and potentially other lymphoid tissues) may perpetuate, at least partially, the pool of functionally exhausted pDCs during infection.

### 4.4. pDC Conversion during Viral Infection

Both acute and chronic LCMV infection as well as the direct administration of PolyI:C or IFN-I drives pDCs to differentiate into cells resembling CD11b^+^ cDCs (cDC2s). These pDC-derived-cDCs exhibit phenotypic and functional features of cDC2s both in vitro [139] and in vivo [140]. While it was first observed in viral infection, pDC conversion has also been reported after stimulation with GM-CSF or intestinal epithelium supernatant [137], in Ly49Q^−^ pDC from PolyI:C injected mice [141], and even after pDC transfer in steady-state conditions [138], suggesting this phenomenon may also regulate pDC numbers at steady state.

Recent subdivision of DC subsets has identified a potential intermediate stage in the transition between pDC and cDC2. High dimensional analysis of the DC compartments of humans and mice identified a “bridge” population termed transitional (t)DCs that occupy a spectrum between pDC and cDC2 [142,143]. This population is formed as an intermediate between pDC and cDC2 like fates in in vitro culture [144], shows intermediate E2-2 expression and IFN-I production when compared to pure pDCs and cDC2, and maps inside the recently described populations of “non-canonical DCs” [5] (i.e., Axl+ DC in humans [142,145] and CX3CR1^+^CD8^+^ cDC in mice [146,147]) that exhibit features of both pDCs and cDCs. Importantly, tDCs accumulate in the lungs of influenza virus infected mice [143]. This is in line with a model where there is increased differentiation from pDC, through tDC, to cDC2 after infection. While these results are in line with a role for pDC differentiation into tDC/cDC2 as a mechanism for reduced pDC numbers during infection, further work will be necessary to definitively show whether the transition between pDC and cDC2 like cells, that was first reported during in vivo viral infection [139,140], uses tDC as an intermediate, and whether disrupting this phenomenon can restore pDC numbers.

## 5. pDCs, IFN-I, SARS-CoV-2, and COVID-19

The COVID-19 pandemic caused by SARS-CoV-2 has led to rapidly evolving investigation of this disease and the virus that causes it. Although pDC have been shown to be protective against beta coronavirus infection in mice [148], the direct protective impact of pDC in SARS-CoV-2 infection has not yet been assessed. Still, much work has been done in a short amount of time to discover how IFN-I regulates SARS-CoV-2 infection and associated COVID-19, how this novel virus is sensed by pDCs, and how infection with SARS-CoV-2 modulates pDC population dynamics and function. Here we summarize the most recent work on these subjects as of the writing of this review.

### 5.1. The Complex Role of IFN-I in COVID-19

The precise role of IFN-I in COVID-19 patients is complex and still emerging. COVID-19 patients that have deficiencies in IFN-I responses as a result of germline mutations or anti-IFN-I autoantibodies are more susceptible to severe disease [149,150,151,152,153]. This is in line with the beneficial role of IFN-I in the control of a variety of viral infections. Furthermore, male identified patients (which typically have low levels of estrogen and only one X chromosome encoding TLR7), are significantly more likely to be hospitalized and develop severe COVID-19 [154], and this could be partly a result of reduced IFN-I production by their pDCs. Notably, use of recombinant IFN-β as a therapeutic for COVID-19 has shown promise in some trials, but not others [155,156,157], and a retrospective study described that early treatment of COVID-19 patients with IFN-α decreased mortality while late treatment resulted in the opposite outcome [158]. In addition, several reports have associated low levels of systemic IFN and reduced IFN-I signatures with severe disease [22,159] while other studies have found positive correlations among IFN-I and severe disease [160,161], although the later may be a consequence of more sustained viral replication that induces more IFN-I in severe cases.

### 5.2. Sensing of SARS-CoV-2 by pDC

It has been established that pDCs can produce IFN-I in response to stimulation with SARS-CoV-2 in vitro [162]. Much work has been done triangulating the PRR used by pDCs to sense SARS-CoV-2. For example, it has been shown that SARS-CoV-2 possesses more ssRNA fragments capable of being recognized by TLR7 and TLR8 when compared to SARS-CoV [163]. Furthermore, a study looking at humans carrying loss of function mutations in UNC93b or IRAK4, which are respectively essential for TLR endosomal localization and signaling [164,165], has shown that their pDCs fail to produce cytokines in response to SARS-CoV-2 exposure [162]. In the same line, SARS-CoV-2, like other coronaviruses, is an RNA virus and TLR7 is essential for pDC sensing of beta-coronavirus in mice [148]. A definitive role for TLR7 in SARS-CoV-2 was recently demonstrated in patients with familial deficiency for TLR7 function. These patients show little to no production of IFN-α2 in their pDCs after stimulation with SARS-CoV-2, though interestingly production of several other inflammatory cytokines was not significantly impacted in this case [153]. Together these studies provide compelling evidence that pDCs sense SARS-CoV2 via TLR7. Consistent with this, familial deficiency in TLR7 leads to increased susceptibility to severe COVID-19 [151,166]. It is important to note that pDC sensing of SARS-CoV-2 is likely to be independent of SARS-CoV-2 infection of pDCs, since human pDCs do not express the SARS-CoV-2 entry receptor ACE2 or support productive replication [162].

### 5.3. Reduced pDC Numbers in SARS-CoV-2 Infection

It is also now well established that, as in other human infections, pDCs are reduced in the peripheral blood of COVID-19 patients [19,20,21,22]. The mechanism underlying this phenomenon has not been fully established, but one study has shown that circulating pDCs from COVID-19 patients have an increased apoptosis signature [22]. As expected, increased pDC apoptosis signatures are negatively correlated with pDC numbers, suggesting increased apoptosis may be responsible for decreased pDC numbers in COVID-19 patients [22]. Intriguingly, increased apoptosis in pDCs also positively correlated with severe disease. Given that pDC numbers also correlate with IFN-I signatures in the same patients, this data is suggestive of a model in which pDC are protective against severe COVID-19, but increased apoptosis limits pDC numbers and IFN-I availability in severe disease [22].

### 5.4. Decreased pDC Function in SARS-CoV-2 Infection

It has been recently shown that pDC from the peripheral blood of COVID-19 patients show reduced IFN-I production in a per-cell basis [19]. Many plausible models exist that might explain this. Several cytokines, including IL-10 and TNFα (discussed above for their potential to suppress pDC function) are elevated in COVID-19 patients [160,167]. Additionally, in COVID-19 patients pDCs show reduced phosphorylation of S6 protein [19], suggesting that mTOR signaling, which supports IFN-I production in pDCs [119], may be compromised in this context. Furthermore, reduction of E2-2 expression upon TLR7 stimulation, as mentioned above for pDC exhaustion in other viral infections [17], is a likely mechanism driving pDC hypo-functionality in COVID-19 patients, although E2-2 levels have not been studied in pDC from SARS-CoV-2 infected cohorts.

### 5.5. Proposed Model for the Biology and Role of pDCs in SARS-CoV-2 Infection

**Figure 1:**
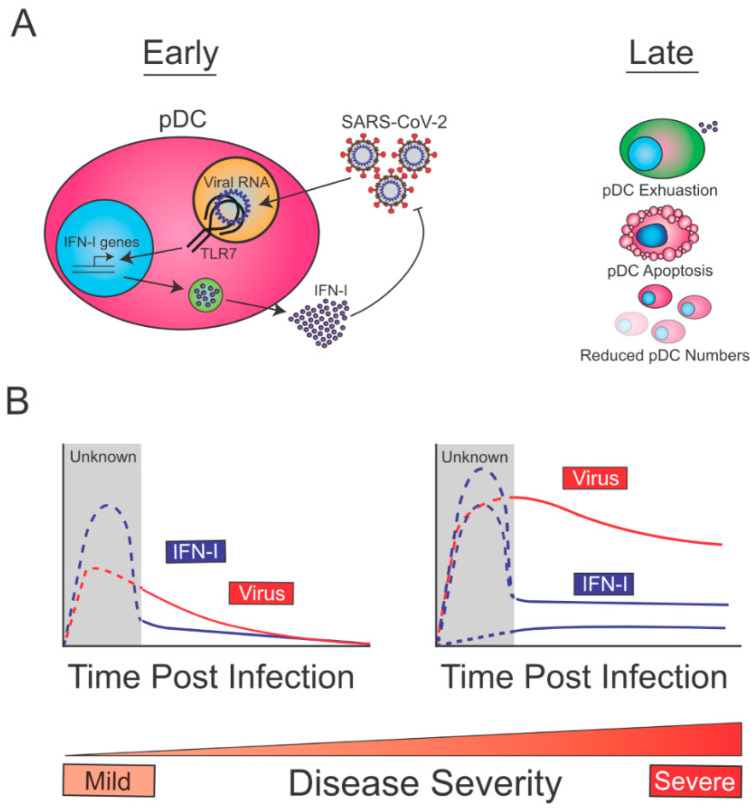
Biology of pDCs during SARS-CoV-2 Infection. (**A**, left) pDC are likely to uptake viral material independent of cell-intrinsic infection, and to sense SARS-CoV-2 RNA via TLR7. This first step stimulates the production of IFN-I which promotes early viral control. (**A**, right) Late after infection blood pDCs become hypofunctional (exhausted), undergo apoptosis and show reduced numbers, and all this may limit pDC derived IFN-I. (**B**, left) pDC from patients with mild disease are expected to produce high IFN-I levels promoting the control of SARS-CoV-2 early after infection. (**B**, right) On the other hand, individuals with severe disease course may exhibit varying degrees of pDC-IFN-I production. The latter may range from low pDC-derived IFN-I in patients with defective TLR7 signaling to normal or even enhanced pDC-derived IFN-I in patients with defects in other immune pathways, or infected with a high viral inoculum or a fast-replicating variant. In such individuals the initial IFN-I wave is expected to be present but insufficient to contain the virus, which may result in sustained stimulation of IFN-I production by a variety of cell types. Importantly, the viral loads or IFN-I levels have not been measured in patients at early times post infection, prior to symptom development, and so the levels of this initial IFN-I response are hypothetical (dotted lines).

Overall, the aforementioned studies are compatible with a model in which, at early times post-infection, pDCs are sensing SARS-CoV-2 through TLR7, which drives the production of IFN-I, and contains SARS-CoV-2 replication (Figure 1A, left). At later time points post-infection, reduction in pDC numbers (exacerbated in severe cases due to increased apoptosis) and decreased pDC function (exhaustion), may lead to reduced pDC-derived IFN-I (Figure 1A, right). Given that early pDC-derived IFN-I is expected to be beneficial for the host to control SARS-CoV-2, this immune function is likely intact in patients with mild disease (Figure 1B, left). On the other hand, COVID-19 severe cases may show diverse profiles of pDC-derived-IFN-I (Figure 1B, right). This may range from defective pDC IFN-I production (e.g., patients with TLR7 loss of function mutations) to intact or even enhanced pDC responses in patients infected with high viral inoculum or fast-spreading variants, or in individuals with deficiencies in other innate defense mechanisms. In the latter patients, sustained viral replication would perpetuate IFN-I levels from a variety of infected cells thereby leading to increased IFN-I signatures. While Arunachalam et al. did not observe any correlation between severity of disease and pDC intrinsic loss of function in COVID-19 patients (albeit with a limited cohort) [19], it is still likely that enhancing or sustaining pDC-derived-IFN-I during the early phase of SARS-CoV-2 infection would help contain the viral spread and favor a rapid resolution of the disease. Future work aiming at better defining the mechanisms by which pDC numbers are reduced and how pDC function is suppressed in COVID-19 patients, may allow us to design therapies to restore/enhance pDC-derived IFN-I early enough to quickly contain viral spread.

## 6. Discussion

IFN-I are powerful cytokines for the control of viruses, but their power also has the potential to harm their host. The diverse mechanisms of IFN-I control in pDCs likely represent an evolved abundance of caution surrounding the power of pDCs to produce IFN-I. Therefore, these suppressive systems are analogous to PD1 expression in CD8 T cells, which prevents autoimmunity and immunopathology, but also favors sustained infections or tumors [168]. They likely represent a natural mechanism of immune regulation more than direct viral strategies to suppress immune responses. This is emphasized most recently in COVID-19 infection where IFN-I can protect the host when administered early [158], and IFN-I deficiencies are associated with severe disease [149,150,151], but, in contrast, late IFN-I treatment may be detrimental [158].

The commonalities that COVID-19 shows with previously-studied infections reemphasizes the importance of the knife’s-edge control that evolution has maintained with interferon. These commonalities also emphasize that the work that has been done understanding IFN-I regulation in other systems offers opportunities to quickly develop strategies to combat novel viral diseases. Beyond viral infection, our understanding of these mechanisms also has implications for the treatment of cancer as pDCs also become exhausted within the tumor microenvironment (Reviewed in [15]). Similarly, for autoimmunity and chronic inflammatory diseases where harnessing of the IFN-I natural braking systems may provide novel therapies. Altogether, the power of pDCs to produce IFN-I is paired by evolution with multiple pathways by which to contain it. By working toward a deeper understanding of those pathways we can harness pDCs for the treatment of a wide spectrum of human illnesses.

## Data Availability

Not applicable.
